# CCAAT/Enhancer-Binding Proteins α and β Regulate Ovulation and Gene Expression via Dose- and Stage-Dependent Mechanisms

**DOI:** 10.1210/endocr/bqaf081

**Published:** 2025-04-28

**Authors:** Hanxue Zhang, Rainer B Lanz, Jimmy Dhillon, Paul D Soloway, Bo Shui, Yi Athena Ren

**Affiliations:** Department of Animal Science, College of Agriculture and Life Sciences, Cornell University, Ithaca, NY 14853, USA; Department of Molecular and Cellular Biology, Baylor College of Medicine, Houston, TX 77030, USA; Department of Biomedical Sciences, College of Veterinary Medicine, Cornell University, Ithaca, NY 14853, USA; Department of Biomedical Sciences, College of Veterinary Medicine, Cornell University, Ithaca, NY 14853, USA; Division of Nutritional Sciences, College of Agriculture and Life Sciences, Cornell University, Ithaca, NY 14853, USA; Department of Biomedical Sciences, College of Veterinary Medicine, Cornell University, Ithaca, NY 14853, USA; Department of Animal Science, College of Agriculture and Life Sciences, Cornell University, Ithaca, NY 14853, USA

**Keywords:** ovulation, transcription factor, C/EBPα, C/EBPβ, gene expression, vascular remodeling

## Abstract

The preovulatory luteinizing hormone (LH) surge orchestrates complex cellular and molecular events leading to ovulation. CCAAT/enhancer-binding proteins α and β (C/EBPα/β) are transcription factors acutely induced by the LH surge and crucial for ovulation and granulosa cell luteinization. However, biological processes (BPs) and their regulatory mechanisms downstream of C/EBPα/β in the preovulatory ovary are not completely understood. To address this knowledge gap, we generated *Cebpa/b^fl/fl^;Pgr-Cre* mutants and compared them with *Cebpa/b^fl/fl^;Cyp19a1-Cre* mutant female mice: *Cebpa/b^fl/fl^;Cyp19a1-Cre* mutants have undetectable levels of C/EBPα/β throughout the preovulatory stages and do not ovulate, aligning with previous reports; and *Cebpa/b^fl/fl^;Pgr-Cre* mutants present gradual depletion of C/EBPα/β during the late preovulatory stage and a reduced ovulation rate. Comparison of these two models indicates that sustained expression of C/EBPα/β throughout the preovulatory stages is necessary for successful ovulation and provides a unique opportunity to interrogate gene regulatory mechanisms by C/EBPα/β during different preovulatory time windows and the effect of dysregulating C/EBPα/β on ovulation-associated BPs. Our study revealed that C/EBPα/β regulate gene expression and distinct biological functions such as vascular remodeling via dose- and preovulatory stage–dependent mechanisms. These findings shed new light on the intricate mechanisms of gene regulation by C/EBPα/β downstream of the LH surge.

The preovulatory luteinizing hormone (LH) surge orchestrates complex cellular and molecular events including oocyte meiotic maturation, granulosa cell (GC) luteinization, the remodeling of ovarian vasculature and extracellular matrix (ECM), and ovarian tissue inflammation (1). These complex processes occur in a temporally controlled manner, culminating in ovulation and subsequent corpus luteum (CL) formation; however, the molecular and cellular mechanisms regulating the spatiotemporal coordination of these processes remain poorly understood.

Specific ovulation-related processes, such as vascularization and inflammation, are precisely regulated to proper activities at distinct stages of the preovulatory period. For instance, vascular endothelial growth factor A (VEGFA) and other angiogenic factors are tightly regulated after the LH surge (2, 3). In rats, the messenger RNA (mRNA) level of *Vegfa* in granulosa cells begins to increase significantly within 4 to 6 hours and peaks around 8 to 12 hours post LH surge. These timely changes correspond to the timing of specific ovarian vascular remodeling processes, such as increased vascular permeability and angiogenesis. The dosage of VEGFA is also critical: Too much or too little can disrupt these processes (4, 5). Another example is endothelin-2, which regulates vasoconstriction and plays a crucial role in ovulation; it is robustly induced immediately prior to ovulation (6-8). The preovulatory stage can be further divided based on the timing of gene induction, with crucial genes being regulated in distinct waves and at multiple preovulatory stages. This is exemplified by the proinflammatory and anti-inflammatory phases observed during the preovulatory period. For example, prostaglandin-endoperoxide synthase 2 (PTGS2), which is induced during the early preovulatory stage (0-6 hours after the LH surge), needs to be downregulated after 6 hours to prevent hyperinflammation, illustrating the importance of timing both for the activation and repression of its expression (9). These observations highlight the complexity and dynamic nature of the preovulatory stage, with both the timing and the level of gene expression finely tuned. Importantly, the control of the timing of events requires both initiating signals and “turning off” signals, and little is known regarding the mechanism of the latter (9).

CCAAT/enhancer binding proteins α and β (C/EBPα/β) are members of the C/EBP family of transcription factors, which are involved in various physiological processes such as immune response, cellular differentiation, and hemostasis (10). C/EBPα and C/EBPβ can form both homodimers and heterodimers, or dimer with other basic leucine zipper domain proteins, which influence their transcriptional outcomes (11). C/EBPβ exists in multiple isoforms from alternative translation initiation, including LAP1 (liver-enriched activator protein 1), LAP2, and LIP (liver-enriched inhibitory protein). LAP1 is the full-length, strong activator of transcription, LAP2 has slightly reduced transcription activity, and LIP acts as a dominant-negative inhibitor of transcription by binding DNA without activating transcription (12). The LAP/LIP ratio often modulates cell differentiation by controlling transcriptional activities (13).

In the ovary, the LH surge induces in GCs the transient expression of C/EBPα and the acute induction and sustained expression of C/EBPβ (14, 15). However, the specific functions of these transcription factors at different preovulatory stages remain unclear. Prior studies have demonstrated that conditional depletion of these two factors in GCs of a transgenic mouse model (*Cebpa/b^fl/fl^;Cyp19a1-Cre* mice, abbreviated as *Cebp-C* mutants) leads to ovulation failure and impaired luteinization, with defective CL structure and reduced expression of luteinization genes (14). However, the cause of ovulation failure in these mutant mice remains to be defined. Furthermore, the depletion of *Cebpa/b* in *Cebp-C* mutants starts as early as the early antral follicle stage when the expression of *Cyp19a1-Cre* becomes detectable (16). This raises the possibility that abnormal biological processes (BPs) associated with ovulation and luteinization in these mutants may have arisen before the preovulatory stage, or are predestined by earlier changes in the differentiation trajectory of ovarian cells. These considerations complicate efforts to determine whether C/EBPα/β directly regulate ovulation or if their effects in *Cyp19a1-Cre* mutants are indirect and primarily consequences of earlier developmental defects.

To address the knowledge gap in whether C/EBPα/β have direct regulatory roles in ovulation and preovulatory gene regulation independent of earlier follicular development, as well as in stage-specific roles of C/EBPα/β in GCs during the preovulatory period, we generated a new transgenic mouse model *Cebpa/b^fl/fl^; Pgr-Cre* (abbreviated as *Cebp-P* mutants), in which the Cre recombinase is expressed after the LH surge (17). This study first characterizes the phenotype of the *Cebp-P* mutants, which exhibit reduced expression of C/EBPα/β during the late preovulatory stage and a reduced rate of ovulation. Comparison between *Cebp-P* mutants*, Cebp-C* mutants, and wild-type (WT) mice provide a unique opportunity to interrogate the function of C/EBPα/β during different stages of the preovulatory period. This study aims to investigate the role and underlying mechanism of C/EBPα/β in ovulation. We identified that C/EBPα/β directly regulate ovulation and preovulatory vascular remodeling in a dose-dependent manner. Additionally, C/EBPα/β regulate gene expression, ovulation-associated BPs, and chromatin accessibility in dose- and preovulatory stage–dependent manners. Our data reveal that C/EBPα/β directly regulate ovulation and unravel the complexity of the underlying mechanisms.

## Materials and Methods

### Animals

*Cebpa/b^fl/fl^;Cyp19a1-Cre* mice (*Cebp-C* mutants) were generated and provided by Dr. JoAnne S. Richards (14). *Cebpa/b^fl/fl^;Pgr1-Cre* mice (*Cebp-P* mutants) were generated by crossing *Pgr-Cre* mice (18) (provided by Dr. John P. Lydon) with *Cebpa/b^fl/fl^* mice. All mutant mouse strains are in the C57BL/6 background. Mice were housed in 11.5″ × 7.5″ IVC Polycarp Shoebox Cage for the duration of the experiment. A temperature of 68 to 77 °F (20-25 °C)and humidity between 30% and 70% were maintained in the rodent room, with lights turned on at 5 Am and off at 7 Pm. All animal work was conducted ethically, conforming to the US Public Health Service policy, and was approved by the Institutional Animal Care and Use Committee at Cornell University (IACUC approved protocol No. 2019-0006).

### Fertility and Ovulation Assessment

The fertility study was conducted by breeding 6-week-old females with fertile C57BL6 males continuously for 6 months (n = 4 for each genotype). Superovulation experiments were performed to assess ovulatory function: female mice aged 21 to 23 days, weighing approximately 10 to 11 g were injected intraperitoneally with 5 IU pregnant mare serum gonadotropin (PMSG, NATE-0969, Creative Enzymes), followed 44 to 48 hours later by the injection of 5 IU human chorionic gonadotropin (hCG, 9002-61-3, Sigma Aldrich). Ovulation efficiency was assessed by counting the number of cumulus-oocyte complexes within both oviducts of superovulated mice at 20 to 24 hours post hCG.

### RNA Extraction, Reverse Transcription, and Real-time Quantitative Polymerase Chain Reaction

GCs were isolated by needle puncture of ovarian follicles. Individual follicles and CL were isolated by manual dissection. Total mRNA from GCs and CL was extracted using an RNeasy Micro Kit (74106, Qiagen). mRNA from individual follicles was extracted using a PicoPure RNA Isolation Kit (KIT0204, Applied Biosystems). Reverse transcription was performed using a High-Capacity cDNA Reverse Transcription Kit (4368814, Applied Biosystems). Real-time quantitative polymerase chain reaction (RT-qPCR) was performed using an RT^2^SYBR Green ROX FAST Mastermix (330623, Qiagen) on StepOnePlus Real-Time PCR System (4376600, Applied Biosystems). Relative levels of mRNA were calculated using the 2(−ΔCt) method and normalized to housekeeping gene *Rpl19*. The primers used are listed in Supplementary Table S1 (19).

### Histology and Immunofluorescence Staining

Ovaries were fixed in 4% paraformaldehyde (15710, Electron Microscopy Sciences) for 4 hours at 4 °C, then washed with phosphate-buffered saline (PBS, QB-119-069-491, Neta Scientific) and stored in 75% ethanol (64-17-5, Fisher Scientific) until dehydration and paraffin embedding, after which 5-μm sections were processed for staining with hematoxylin and eosin or for immunofluorescence staining. For staining of C/EBPβ, paraffin sections were deparaffinized and autoclaved in citric acid buffer (pH 6.0). Sections were blocked with 2% bovine serum albumin (BSA, BP9700100, Fisher scientific)-PBS containing 0.1% Tween 20 for 1 hour at room temperature and then incubated with primary antibody (anti-CEBP β [E299], Abcam catalog No. ab32358, RRID:AB_726796 at 1:100) overnight at 4 °C. Sections were washed and incubated with secondary antibody (Goat Anti-Rabbit IgG (H + L) conjugated with Alexa 594, Molecular Probes catalog No. A-11012, RRID:AB_141359) at 1:1000 dilution for 1 hour at room temperature, followed by cell nucleus counterstaining with 1 μg/mL Hoechst 33342 (76482-876, VWR). Images were obtained using Nikon Diaphot 300 microscope (Nikon Instruments) and processed with ImageJ.

### Western Blotting

GCs were lysed in RIPA Lysis buffer (BP-115, Boston BioProducts). Proteins of different molecular weight were separated using a 10% Bis-Tris gel (Bio-Rad, 4569033) and transferred to an immobilon-P membrane (Millipore). Membranes were blocked with 3% BSA-TBST for 1 hour at room temperature and then incubated with primary antibodies (anti-CEBP β [E299] at 1:1000 dilution; anti-C/EBP α, Proteintech catalog No. 18311-1-AP, RRID:AB_2077892 at 1:1000 dilution) overnight at 4 °C. Membranes were washed and incubated with corresponding secondary antibodies (goat Anti-Rabbit IgG (H + L) HRP, Abcam, catalog No. ab7090, RRID:AB_955417) at 1:5000 dilution for 1 hour at room temperature. Images were taken using a laser fluorescence scanner (LI-COR), processed, and quantified with ImageJ (2.9.0/1.54, RRID:SCR_003070).

### Whole-mount Staining and Imaging

At approximately 12 hours post hCG, immediately prior to ovulation, the mice underwent anesthesia by isoflurane, and the ovarian vasculature was labeled by retro-orbital injection of 10 μL DyLight 649-labeled Lycopersicon Esculentum (Tomato) Lectin (LEL, TL) (L32472, ThermoFisher). Mice were euthanized by CO_2_ inhalation 5 minutes after lectin injection. Ovaries were collected and fixed in 4% paraformaldehyde at 4 °C for 4 hours. Fixed ovaries were then washed 3 times in PBS for 10 minutes each before tissue clearing using CUBIC1/2 as previously described (20). Ovaries were cleared in CUBIC-1 solution by gentle rocking at room temperature for 4 to 5 days. After washing 3 times in PBS for 10 minutes each, ovaries were incubated in 20% sucrose-PBS overnight. Subsequently, cell nuclei were stained with Hoechst 33258 in CUBIC-2 solution for at least 24 hours with gentle rocking at room temperature. Last, ovaries were washed using CUBIC-2 solution for 4 hours and stored in fresh CUBIC-2 solution at room temperature until imaging.

Ovaries were imaged using an inverted multiphoton laser scanning confocal microscope (Zeiss LSM880 microscope). Images were taken at 10-μm intervals starting from the outer surface of the ovary to generate a 550- to 600-μm-thick z-stack. Z-stack images were reconstituted into 3-dimensional (3D) projection images in Arivis 4D (RRID:SCR_018000). Periovulatory follicles were identified based on the presence of expanded cumulus-oocyte complexes and an intact follicle wall. Ovarian vascular structures were carefully examined with attention to the stromal vessels surrounding the periovulatory follicles. Furthermore, clearing of the capillary beds at the apex (follicle rupture site) of periovulatory follicles was assessed; where the apex was not parallel to the imaging plane, we rotated the reconstituted 3D images in Arivis 4D to better visualize the structure of the capillary bed.

### Bulk RNA Sequencing and Data Analysis

Total mRNA was extracted from GCs using the RNeasy Micro Kit according to the manufacturer's instructions. GCs isolated from 1 ovary of 1 mouse were used as 1 replicate, and 3 independent biological replicates (n = 3 mice) were used for RNA sequencing (RNA-seq) library generation. Library preparation and transcriptome sequencing were conducted by Novogene Co, Ltd. Raw data (raw reads) of fastq format were first processed through fastp software. In this step, clean data (clean reads) were obtained by removing low-quality reads and reads containing adapter and ploy-N from the raw data. At the same time, Q20, Q30, and GC content of the clean data were calculated (Supplementary Table S2 (19)). All the downstream analyses were based on the clean data with high quality. Paired-end clean reads were aligned to the mouse genome (GRCm38/mm10) using HISAT2 (version 2.0.5) by default parameters. Read alignment rates and percentage of genome regions of each sample are shown in Supplementary Fig. S1 (19). Read counts for each gene were generated using featureCounts (v1.5.0-p3), and transcripts per million (TPM) values of each gene were generated using EdgeR (version 1.3.3). Only genes expressed in at least one replicate with TPM greater than 1 were included for further bioinformatics analyses. DESeq2 R package (version 1.20.0) was used for differential gene expression analyses with cutoff fold changes greater than 2 and *P* values less than .05 (Supplementary Tables S3 and S4 (21, 22)). Differentially expressed genes (DEGs) were visualized using VolcaNoseR (23). Comparisons of DEGs between *Cebp-C* and *Cebp-P* mutants were visualized using VennDetail Shiny App (http://hurlab.med.und.edu:3838/VennDetail/). The TPM values of each gene were first transformed by robust *Z*-score method using R, then used as the input for K-means clustering and heatmap plotting by Phantasus (https://artyomovlab.wustl.edu/phantasus/). Gene Ontology (GO) analyses were performed using the online tool Metascape (24) (http://metascape.org) and were visualized using Hiplot (ORG) (25) (https://hiplot.org).

### Single-Nucleus Assay for Transposase-accessible Chromatin Sequencing

Single-nucleus assay for transposase-accessible chromatin sequencing (snATAC-seq) was conducted following a previously described protocol with some modification for ovarian tissues (26).

### Expression and Purification of Homemade Tn5

We used an adapted protocol based on the method by Hennig and colleagues (27) for homemade Tn5. The Tn5 cassette, PCR-amplified from pTXB1-Tn5, was subsequently subcloned into the pET-SUMO vector with the following orientation: 5′ BamHI (ATG)-Tn5-(TAA) HindIII 3′. The resulting pET-SUMO-Tn5 plasmid was transformed into BL21 Star (DE3) pLysS E. coli. Cells were cultured in TB/LB (1:1) supplemented with kanamycin and chloramphenicol at 37 °C until reaching an OD600 of approximately 0.5. Subsequently, the temperature was reduced to 18 °C to 20 °C and expression of Tn5 was induced with 0.2 mM IPTG. After overnight growth at 18 °C to 20 °C, cells were harvested by centrifugation. The cell pellets were resuspended in binding buffer (20 mM HEPES-NaOH pH 7.2, 800 mM NaCl, 20 mM imidazole, 0.2 mM EDTA, 2 mM DTT, 10% glycerol, and 0.2% Triton-X100) with complete protease inhibitors and lysed by sonication. The insoluble debris was pelleted by centrifuging the sonicated bacteria at 16 000*g* at 4 °C for 30 minutes. Polyethyleneimine pH 7.2 was added dropwise to a final concentration of 1% for nucleic acid removal, then centrifuged at 16 000*g* at 4 °C for 30 minutes. The supernatant was incubated with 2 mL Ni-charged Profinity IMAC Resin for 1 hour with shaking. The mixture was loaded onto 10-mL Pierce Centrifuge Columns for gravity flow-through. The mixture was washed 5 times with 8 mL of binding buffer. The His-SUMO-Tn5 was eluted in 6 mL binding buffer containing 300 mM imidazole, followed by an additional 6 mL of binding buffer. To remove the fusion tag, homemade His-tagged-ulp-1 proteinase was added to the elution fractions. The samples were loaded into Slide-A-Lyzer G2 Dialysis Cassettes (10 K MWCO, 15 mL) and digested overnight at 4 °C while being dialyzed back to the binding buffer. To eliminate His-SUMO and His-ulp-1, the dialyzed sample was bound to 2 mLNi-charged Profinity IMAC Resin, loaded onto 10-mL Pierce Centrifuge Columns, and the untagged Tn5 was collected in the flow-through by gravity. Concentration and buffer exchange were performed using Amicon Ultra-15 Centrifugal Filter Units (30 K) and transitioned to the storage buffer (100 mM HEPES-KOH at pH 7.2, 0.2 M NaCl, 0.2 mM EDTA, 2 mM DTT, 0.2% Triton X-100, 20% glycerol) (28). The concentration was determined using the Pierce 660 nm Protein Assay. The Tn5 concentration was adjusted to 5 µM using the storage buffer and stored at −80 °C.

### Transposome Assembly

Tn5 transposase (5 μM) was diluted by adding 1 volume of 80% glycerol, resulting in a concentration of 2.5 μM. Subsequently, Tn5 transposome were assembled by adding 0.1 volume of Tn5 adaptors (25 μM) to the Tn5 (2.5 μM), and the mixture was incubated at room temperature overnight. The assumed concentration of the transposomes was 2 μM.

### Tagmentation and Sample Processing

The nuclei extraction protocol employed the same lysis buffer (1× homogenization buffer) and 1× washing buffer (1× ATAC-RSB + 0.1% Tween-20), as described by Corces et al (29). The modification in the extraction steps includes two homogenization stages: initially in 1× homogenization buffer with a loose pestle, followed by the second homogenization step in 1× washing buffer. Nuclei suspension (8 μL) was distributed onto a 96-well plate and 3 μL of 2 barcoded transposomes were added to each well. Each well contained approximately 5000 nuclei and 400 µM transposomes in 1× TD buffer (10 mM Tris-HCl, pH 7.6, 5 mM MgCl2, 10% DMF, 0.33× PBS, 0.1% Tween20, 0.01% Digitonin). The plate was incubated for 30 minutes at 50 °C and the reaction was terminated by adding 10 μL 40 mM EDTA to each well. All nuclei were combined into a single sample, and 2 mL of sorting buffer (1× PBS, 0.2% BSA, 2 mM EDTA) was added. Intact nuclei were then stained with 20 μL of Draq7 (ab109202, Abcam) on ice for 15 minutes, filtered through a 30-μm filter, and sorted by fluorescence-activated cell sorter using a Cell Sorter (BD FACSMelody). Twenty-five nuclei were distributed into each well of a 96-well plate preloaded with 10 μL Sort EB (10 mM Tris-HCl, pH 8.0, 0.05% sodium dodecyl sulfate), and incubated for 15 minutes at 50 °C to lysis nuclei and finish tagmentation. A total of 2.5 μL of 5% Triton-X100 was added into each well to quench sodium dodecyl sulfate prior to PCR.

### Polymerase Chain Reaction Amplification (Library Generation) and Size Selection

For each well in a 96-well plate, the 12.5 μL of 2× PCR reaction master mix contains 5 μL Q5 Reaction buffer, 5 μL High GC Enhancer, 0.5 μL dNTP mix (10 mM), 0.25 μL Q5 DNA Polymerase, 0.5 μL Universal P5 primer (25 μM), 0.75 μL Nuclease-Free Water; and a barcoded i7 primer was added. Libraries were amplified using the following program: 72 °C for 5 minutes, 98 °C for 30 second, then 14 cycles of (98 °C for 15 second, 66 °C for 30 second, 72 °C for 40 second). PCR products from all wells were repooled and purified using QIAquick spin columns, followed by 2 rounds of size selection with AMPure XP beads. The first round was at 0.5×/1.2× and the second round was at 1.2×. Finally, the DNA was eluted in 40 μL of 10 mM Tris, pH 8.0.

### Library Quantification, Quality Control, and Sequencing

The concentration of libraries was measured using the Qubit. DNA fragment length distribution was analyzed by Bioanalyzer High Sensitivity DNA Chip (Agilent Genomics). Libraries were sequenced using the Illumina HiSeq X platform.

### Preprocessing and Analysis of Single-Nucleus Assay for Transposase-Accessible Chromatin Sequencing Data

Undemultiplexed fastq files were processed using cutadapt (30), UMI tools (31), and custom scripts (26) to parse and assemble combinatorial barcodes from read segments and extract valid single-nucleus barcodes (with error correction). Adapter sequences were trimmed using cutadapt (version 4.1). Alignment to the mm10 reference genome was performed using bowtie2. Mitochondrial reads and reads mapped to blacklisted regions were then removed. Duplicate reads were removed using umi_tools dedup (version 1.1.4). Identification of cell clusters and cell types were performed in ArchR (32). We initially obtained 15 383 cells after filtering out cells that had fewer than 1000 fragments and a transcription start site (TSS) enrichment score of less than 4. After filtering out doublets, there were 15 091 cells with a median TSS score of 12.973 and a median number of fragments of 8042. After the initial quality check, IterativeLSI (Latent Semantic Indexing) was added to the ArchR project. The data were then clustered using the default resolution of 0.8. The clusters were visualized using uniform manifold approximation and projection (UMAP). A total of 14 clusters (Supplementary Table S5 (33)) were identified, and cell types were assigned using marker genes from the existing literature (9, 34). To visualize genes of interest, gene scores were imputed using MAGIC. Genes of interest were then visualized on the UMAP. To visualize coaccessibility, peaks were first called using MACS2 and a peak matrix was added to the ArchR project. Marker peaks were identified, and coaccessibility was calculated using the “addCoAccessibility” function. Browser tracks were plotted for the clusters of interest (clusters 1-4) for the selected genes of interest.

### Pan-Cistromics Analysis

DNA-binding experiments curated by the ReMap2022 project (35) presented as nonredundant peaks for transcriptional regulators (TRs) across all experiments or provided as cis regulatory modules (CRMs) detecting peak density along the genome were downloaded for the *mus musculus* GRCm39/mm39 assembly and used in two different Claris FileMaker Pro solutions. To analyze any number of genes for TRs binding to a specified gene boundary, *mus musculus* gene annotations GCF_000001635.27_GRCm39_feature_table.txt.gz were downloaded from NCBI (https://www.ncbi.nlm.nih.gov/datasets/genome/?taxon=10090), the data were filtered for feature_type “GENE,” and the genomic annotations were recalculated for the extended promoter region (EPR: TSS—7.5Kb, TSS + 2.5KB) for all genes. These EPRs of gene annotations were intersected with the ReMap 2022 CRM coordinates using Galaxy “Operate on Genomic Intervals” to produce a join table that shows all TRs that bind to the extended promoter region of all official NCBI gene annotations. To retain TRs and biotype information for any genomic position, the ReMap2022 nonredundant peak set was related with query intervals (Int) via constraints on chromosomes and the following coordinates: chrPeak = chrInt AND PeakStart < IntEnd AND PeakEnd > IntStart.

TRs from the ReMap 2022 CRM that bind within the EPR of the selected genes are listed in Supplementary Table S6 (36) (“EPR_selGenes” worksheet). Genomic coordinates of peaks for C/EBPα or C/EBPβ binding with their associated biotype data from the ReMap 2022 nonredundant peaks set are provided for the genomic loci (official gene annotations expanded to include transcription permissive regulatory regions as annotated by ENCODE Candidate Cis-Regulatory Elements) of the selected genes in the “CEBP_selGenes” worksheet). All coordinates are based on the GRCm39/mm39 mouse genome assembly. UCSC's Lift Genome Annotations (https://genome.ucsc.edu/cgi-bin/hgLiftOver) was used to convert (GRCm39/mm39) coordinates to GRCm38/mm10 annotations to show the overlapping intervals in Supplementary Table S7 (37).

### Statistical Analysis

All quantitative data are presented as mean ± SD. Statistical analysis was performed using GraphPad Prism 10 analysis software (RRID:SCR_002798). Each experiment included at least 3 independent biological samples and was repeated at least 3 times. Cross-sample comparisons between 2 groups were made using the 2-tailed unpaired *t* test. For the statistical analysis in Fig. 2D, the *t* test was conducted using arcsine square root-transformed data to account for the distribution of percentage values. For comparison across 3 or more groups, 1-way analysis of variance (ANOVA) or 2-way ANOVA followed by the Tukey multiple comparisons test were used. A *P* value less than .05 was considered statistically significant.

## Results

### Conditional Depletion of *Cebpa/b* at Different Stages of Follicle Growth by *Cyp19a1-Cre* vs *Pgr-Cre* Led to Different Ovulation Outcomes

**Figure 1.**
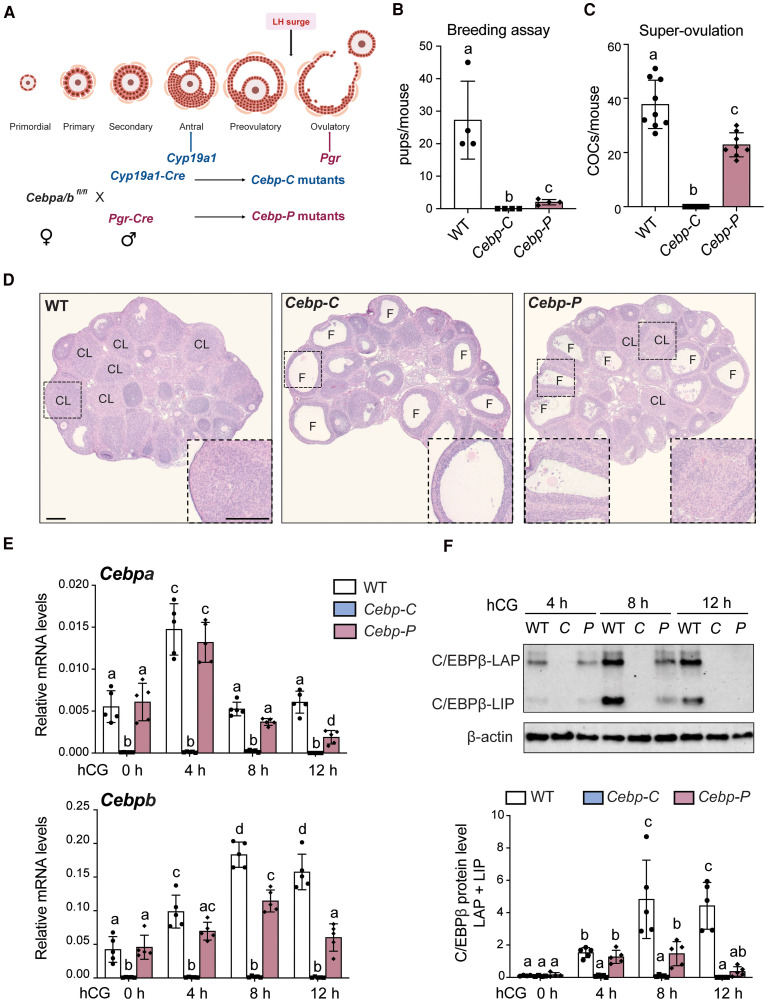
Conditional depletion of *Cebpa*/*b* at different stages of follicle growth by *Cyp19a1-Cre* vs *Pgr-Cre* led to different ovulation outcomes. A, Schematic diagram of follicle development and the generation of *Cebp-C* and *Cebp-P* mutants. The timing of expression for each Cre recombinase is indicated in the diagram. B, Breeding assay of WT, *Cebp-C*, and *Cebp-P* mutants (n = 4). C, Superovulation studies of WT, *Cebp-C*, and *Cebp-P* mutants (n = 8-9). D, Hematoxylin-eosin staining shows the ovarian histology of WT, *Cebp-C*, and *Cebp-P* mutants at 24 hours post hCG. Scale bar = 200 μm. CL, corpus luteum; F, follicle. E, RT-qPCR shows the mRNA levels of *Cebpa* and *Cebpb* in GCs collected from WT, *Cebp-C*, and *Cebp-P* mutants before and after hCG treatment (n = 5). F, Western blot shows C/EBPβ levels in GCs collected from WT, *Cebp-C*, and *Cebp-P* mutants after hCG treatment (n = 5). Quantitative data in this figure are presented as mean ± SD. B and C, One-way ANOVA and E and F, 2-way ANOVA with Tukey multiple comparisons test were used for statistical analysis. Bars without common superscripts are statistically different (*P* < .05).

To investigate the function of C/EBPα/β between the LH surge and ovulation, we compared WT mice and 2 genetically modified mouse models in which *Cebpa*/*b* were conditionally depleted using *Cyp19a1-Cre* (*Cebp-C* mutants) and *Pgr-Cre* (*Cebp-P* mutants), respectively (Fig. 1A). The expression of *Cyp19a1* initiates in GCs at the early antral follicle stage (16), whereas *Pgr* is primarily expressed in the mural GCs of preovulatory follicles after the LH surge (9) (Fig. 1A). Consistent with previously published data (14), *Cebp-C* mutants failed to produce offspring when bred with fertile males for 6 months (Fig. 1B) and exhibited blocked ovulation following superovulation (Fig. 1C). In comparison, *Cebp-P* mutants were nearly infertile (see Fig. 1B) and exhibited a 40% reduction in ovulation rate compared to WT mice when superovulated (37.78 ± 8.941 vs 22.88 ± 4.454; *P* < .05; see Fig. 1C). *Pgr-Cre* is also expressed in the uterus (38), and C/EBPβ has been shown to play a role in decidualization during pregnancy (39). This may explain why the fertility defects in *Cebp-P* mutants were more severe than can be explained by the ovulation defects. The ovarian histology of WT and the 2 mutants at 24 hours post hCG is consistent with their respective rates of ovulation, demonstrating a number of well-formed CL in WT, no CL but large unruptured follicles with entrapped oocytes in *Cebp-C* mutants, and the presence of both unruptured follicles with entrapped oocytes and CL in *Cebp-P* mutants (Fig. 1D).

Based on the differential expression timeline of the *Cyp19a1-Cre* and *Pgr-Cre*, we hypothesized that the difference in ovulation rates between the 2 mutants could be attributed to the timing of C/EBPα/β protein depletion by the respective Cre recombinases. To test this hypothesis, we assessed the expression levels of mRNAs and proteins of *Cebpa*/*b* using RT-qPCR and Western blot. Consistent with previous reports (14, 15), in WT mice, both *Cebpa*/*b* mRNA and proteins were induced by hCG stimulation (Fig. 1E and 1F and Supplementary Fig. S2A and S2B (19)). Specifically, *Cebpa* mRNA and protein levels peaked at 4 hours post hCG and then decreased to the baseline level as at 0 hours post hCG (see Fig. 1E and Supplementary Fig. S2A (19)). In contrast, *Cebpb* mRNA and protein levels peaked at 8 hours and were sustained until at least 12 hours post hCG (ovulation occurs ∼12 hours post hCG in mice) (see Fig. 1E and 1F). In *Cebp-C* mutants, *Cebpa/b* mRNA and protein levels were barely detectable following hCG stimulation (see Fig. 1E and 1F and Supplementary Fig. S2A (19)). In comparison, in *Cebp-P* mutants, the mRNA levels of *Cebpa* began to decrease at 8 hours post hCG, with protein levels following suit at 12 hours post hCG. In contrast, *Cebpb* mRNA and protein levels both remained at normal levels until at least 4 hours post hCG (see Fig. 1E and 1F). Interestingly, while the protein levels of C/EBPβ doubled between 4 to 8 hours in WT mice, it stayed constant in *Cebp-P* mice until declining to a basal level by 12 hours (see Fig. 1F). However, in *Cebp-P* mutants, *Cebpa/b* mRNA and protein levels were both still detectable and were higher than those in *Cebp-C* mutants until 12 hours post hCG (see Fig. 1E and 1F and Supplementary Fig. S2A (19)), indicating the incomplete depletion of *Cebpa/b* in *Cebp-P* mutants before ovulation. Additionally, given the distinct effects of C/EBPβ-LAP as an activator and C/EBPβ-LIP as a dominant-negative inhibitor of gene transcription, we sought to quantify changes in the ratio of these isoforms post hCG treatment to better understand how the ratio between these isoforms changes during the preovulatory period. The ratio of C/EBPβ-LAP to -LIP isoforms decreased from 4 to 8 hours, followed by a slight increase from 8 to 12 hours post hCG in WT (Supplementary Fig. S2C (19)), supporting active regulation of this ratio and its potential involvement in preovulatory gene regulation. In *Cebp-P* mutants, the LAP/LIP ratio also declined between 4 to 8 hours but was higher than in WT (see Supplementary Fig. S2C (19)), suggesting that the relative abundance of the activator isoform LAP is higher in *Cebp-P* mutants than WT at 8 hours post hCG, potentially contributing to dysregulation of gene expression in these mutants.

Taken together, these results indicate that normal expression of C/EBPα/β during the early post hCG period (at least the first 4 hours) is critical but not sufficient for normal ovulation, and the subsequent increase and maintenance of C/EBPβ expression beyond 4 hours are also required.

### Expression Levels of *Cebpa/b* in Individual Preovulatory Follicles Are Correlated With Ovulation Outcome

**Figure 2.**
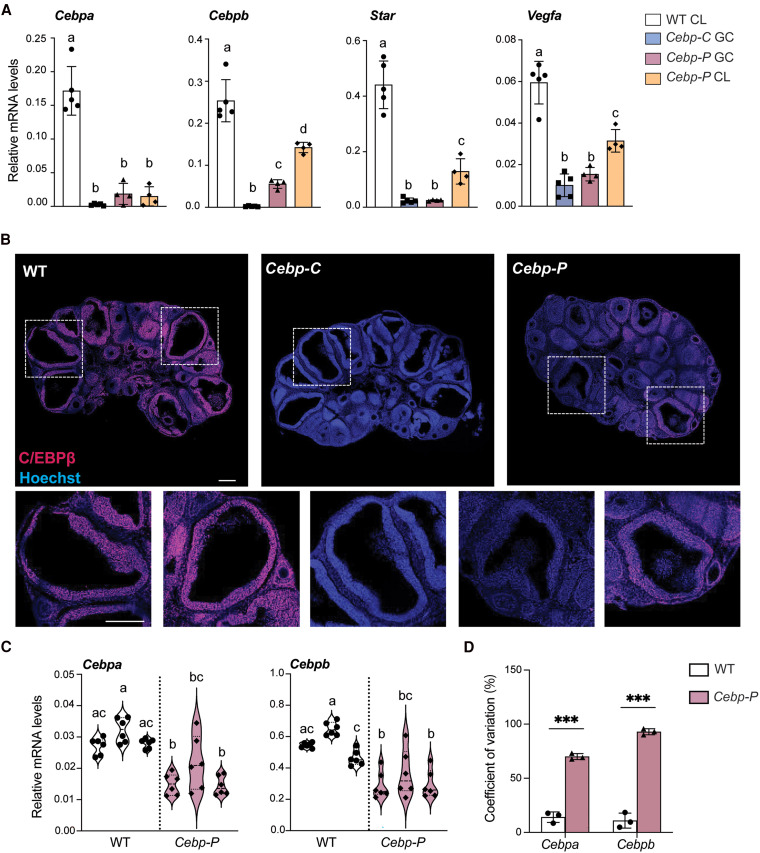
The expression levels of *Cebpa/b* in individual preovulatory follicles are correlated with ovulation outcome. A, RT-qPCR shows the mRNA levels of selected genes in GCs and CL collected from WT, *Cebp-C*, or *Cebp-P* mutants at 24 hours post hCG (n = 5). B, Immunofluorescence staining of C/EBPβ in mouse ovaries of WT, *Cebp-C*, and *Cebp-P* mutants at 8 hours post hCG (n = 3-4). Scale bar = 200 μm. C, RT-qPCR shows the mRNA levels of *Cebpa* and *Cebpb* in individual follicles collected from WT and *Cebp-P* mutants at 8 to 9 hours post hCG (n = 3). Dots in the same column are from the same ovary. D, Coefficient of variation analysis in C. Quantitative data in this figure are presented as mean ± SD. One-way ANOVA with Tukey multiple comparisons test was used for statistical analysis in A and C (left and middle panel). Multiple two-tailed unpaired *t* test was used for statistical analysis in C and D. Arcsine square root-transformed data in D were used to meet the assumptions of the *t* test. Bars without common superscripts are statistically different (*P* < .05). ***P < .001.

We hypothesized that the outcome of follicle rupture in *Cebp-P* mutants is sensitive to the extent of *Cebpa/b* depletion in individual follicles. This hypothesis arises from our observations in Fig. 1, which shows that *Cebpa/b* depletion in *Cebp-P* mutants begins around 4 hours post hCG, with their expression persisting until shortly before ovulation. By 24 hours post hCG, while WT mice have formed CL from ruptured follicles, no CL formation was observed in *Cebp-C* mutants, and *Cebp-P* mutants exhibited both unruptured follicles and CL (see Fig. 1D). We were curious why some follicles in *Cepb-P* mutants ovulate but other do not and hypothesized that this may be linked to the variable extent of *Cebpa/b* depletion in individual follicles. To test this hypothesis, we isolated CL from WT, GCs from unruptured large antral follicles from *Cebp-C* mutants, and both GCs (from unruptured large antral follicles) and CL from *Cebp-P* mutants at 24 hours post hCG (early luteal stage). Subsequently, we compared the mRNA levels of *Cebpa/b* and genes essential for CL formation and function across these 4 groups (Fig. 2A). Compared to WT CL, mRNA levels of *Cebpa* were comparable across the other 3 groups and at a significantly lower level; mRNA levels of *Cebpb* varied across the other 3 groups, albeit all lower than WT CL. These data suggest uncoupling of mRNA levels of *Cebpa* and *Cebpb* at the early luteal stage. More importantly, these data suggest that a threshold mRNA level of *Cebpb* for successful follicle rupture appears to be between 30% (no follicle rupture) to 60% (follicle rupture) of the level in normal mice (*Cebp-P* GC vs *Cebp-P* CL). The mRNA levels of the steroidogenic gene *Star* (40) and angiogenic gene *Vegfa* (41) were also lower in GC and CL from the 2 mutant mouse lines compared to controls, with CL from *Cebp-P* mutants having higher mRNA levels of *Cebpb*, *Star*, and *Vegfa* compared to GCs from unruptured follicles. These results suggest that where follicle rupture fails to occur (*Cebp-C* GC and *Cebp-P* GC) with below 30% of the normal transcript level for *Cebpb*, steroidogenesis and angiogenesis genes have severely reduced mRNA levels and are not sensitive to further reduction of *Cebpb* mRNA levels. In addition, despite successful follicle rupture (WT CL vs *Cebp-P* CL), reduced mRNA levels of *Cebpb* corresponded with reduced mRNA levels of *Star* and *Vegfa*, suggesting a direct and dose-sensitive role of C/EBPβ in regulating the expression of these genes in the early CL. Based on these data, it is unclear to what extent the outcome of follicle rupture vs the level of *Cebpb* mRNA contribute to the mRNA levels of *Star* and *Vegfa* as the level of *Cebpb* mRNA is also correlated with follicle rupture (*Cebp-C* GC, *Cebp-C* GC vs *Cebp-P* CL). Taken together, these observations support our hypothesis that the outcome of follicle rupture is sensitive to the extent of *Cebpb* depletion in individual follicles.

Considering the potential direct role of C/EBPα/β in regulating gene expression in CL in addition to its effects on ovulation, the interpretation of the aforementioned data may be complicated as they were collected in the early luteal phase. Therefore, we further compared gene expression in GCs from ruptured and unruptured follicles at 14 hours post hCG immediately after the anticipated time for ovulation in normal mice (Supplementary Fig. S3 (19)). These data at 14 hours post hCG mostly showed a similar trend of difference across groups and supported our interpretations of data in Fig. 2A, with a few minor but interesting additional observations: mRNA levels of *Cebpa* differed across all 4 groups; mRNA levels of *Vegfa* differed between *Cebp-C* unruptured F and *Cebp-P* unruptured F; and the mRNA levels of both *Cebpa* and *Vegfa* correlated with the mRNA levels of *Cebpb*, suggesting dose-dependent regulation of their transcription by *Cebpb*. The expression of *Cebpa* was reported to decrease over time after ovulation in normal mice (14), which may be a result of a decreasing level of transcripts that accounts for the convergence across groups by 24 hours post hCG. These data together further support that the outcome of follicle rupture is sensitive to the extent of *Cebpb* depletion in individual follicles. If this is the case, at the individual follicle level, there would be varying levels of expression of *Cebpa*/*b* in *Cebp-P* mutants. Indeed, immunostaining of C/EBPβ at 8 hours post hCG showed that C/EBPβ protein was predominantly expressed in GCs of preovulatory follicles and preantral follicles in WT but was undetectable in *Cebp-C* mutants (Fig. 2B). As anticipated, the staining signal of C/EBPβ in *Cebp-P* mutants appeared weaker overall compared to that in preovulatory follicles of WT mice, and varied across different preovulatory follicles, with some follicles displaying a minimal signal similar to that in *Cebp-C* mutants while other follicles displayed an appreciable signal (see Fig. 2B). The level and variation of *Cebpa*/*b* mRNA across different preovulatory follicles of *Cebp-P* mutants were further quantified in isolated individual preovulatory follicles from WT and *Cebp-P* mutants at 8 to 9 hours post hCG. Consistent with immunostaining, mRNA levels of *Cebpa*/*b* displayed a larger variation across different preovulatory follicles from the same *Cebp-P* mutant mice compared to WT mice (Fig. 2C and 2D). Interestingly, the average levels of *Cebpb* mRNA in GCs varied across WT mice (see Fig. 2C), suggesting a potential correlation of *Cebpb* expression with the physiological state of individual mice. Despite the variation among individual follicles in the *Cebp-P* mutants, there was an overall trend of decreased levels of mRNA both for *Cebpa* and *Cebpb* in the preovulatory follicles of *Cebp-P* mutants compared to WT mice (see Fig. 2C). This suggests that while some follicles in *Cebp-P* mutants may retain near-normal levels of *Cebpa*/*b*, others experience substantial downregulation, contributing to the variability in follicle rupture and CL formation. Taken together, these data indicate that the depletion of *Cebpa*/*b* by *Pgr-Cre* is uneven across individual preovulatory follicles, and that the extent of their depletion is correlated with the outcome of follicle rupture.

### C/EBPα/β Regulate Biological Processes Critical to Ovulation via Dose- and Preovulatory Stage–Dependent Mechanisms

**Figure 3.**
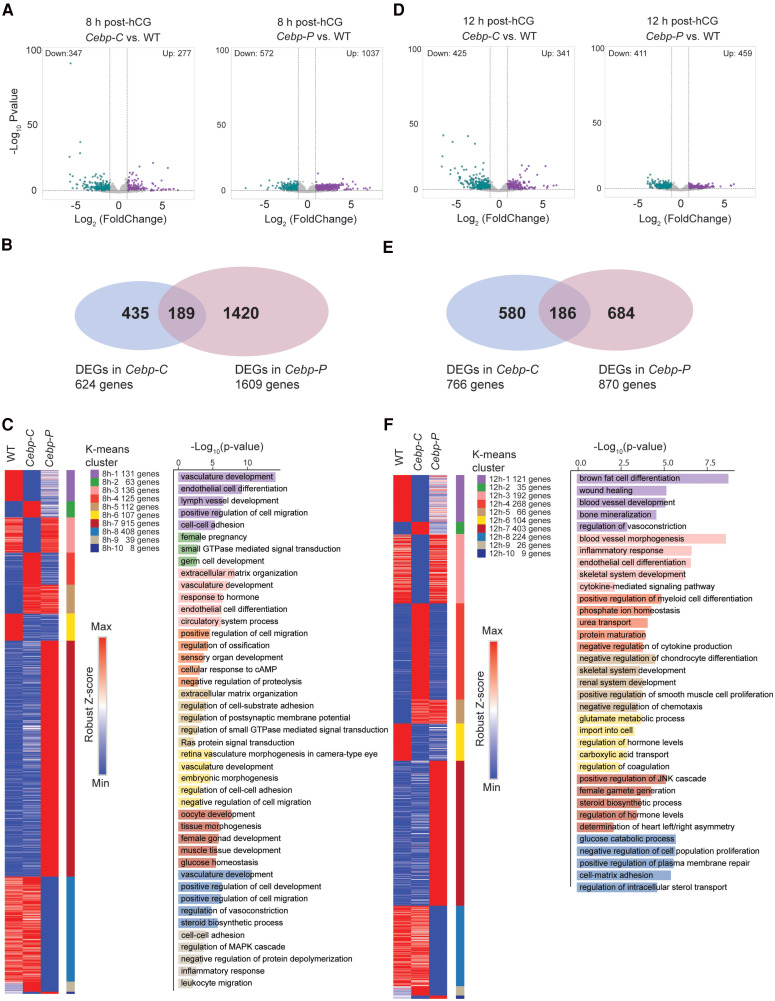
C/EBPα/β regulate biological processes critical to ovulation via dose- and preovulatory stage–dependent mechanisms. A, Volcano plots show the DEGs in GCs from *Cebp-C* (left panel) and *Cebp-P* (right panel) mutants to WT at 8 hours post hCG (n = 3) (DEGs: log2 fold change >1; *P* < .05). B, Venn diagram shows the overlap between DGEs of *Cebp-C* and *Cebp-P* mutants in A. C, Heat maps show K-means clustering of all DEGs in B. The colors in the map represent robust *Z* Score–normalized values for DEGs: Blue indicates the lowest expression, white indicates intermediate expression, and red indicates the highest expression. The genes of each cluster were subjected to a biological process GO enrichment analysis, and the top enriched GO terms for each cluster are shown. D, Volcano plots show the DEGs in GCs from *Cebp-C* (left panel) and *Cebp-P* (right panel) mutants to WT at 12 hours post hCG (n = 3). E, Venn diagram shows the relationship between DGEs of *Cebp-C* and *Cebp-P* mutants in D. F, Heat maps show K-means clustering of all DEGs in E. The genes of each cluster were subjected to a biological process GO enrichment analysis, and the top enriched GO terms for each cluster are shown.

Given that *Cebp-C* and *Cebp-P* mutants have different timelines and extents of depletion of *Cebpa*/*b*, and differentially impaired ovulation (blocked ovulation in *Cebp-C* mutants; reduced ovulation in *Cebp-P* mutants), comparing DEGs and affected BPs between the 2 mutants may inform molecular and cellular processes important for ovulation during specific time windows of the preovulatory stage, and how these processes are regulated by C/EBPα/β. To this end, we conducted bulk RNA-seq analysis on GCs isolated from WT, C*ebp-C*, and *Cebp-P* mutants at 8 hours and 12 hours post hCG. At 8 hours post hCG, compared to WT, *Cebp-C* mutants exhibited 277 upregulated and 347 downregulated genes, and *Cebp-P* mutants displayed 1037 upregulated and 572 downregulated genes (Fig. 3A; Supplementary Table S3 (21)). We further compared these DEGs relative to WT between *Cebp-C* and *Cebp-P* and found that 189 DEGs overlapped between *Cebp-C* and *Cebp-P* mutants, 435 DEGs were specific to *Cebp-C* mutants, and 1420 DEGs were specific to *Cebp-P* mutants (Fig. 3B). Interestingly, the top enriched BPs in all 3 groups (shared DEGs, *Cebp-C*–specific DEGs, and *Cebp-P*–specific DEGs) were related to vascular remodeling (Supplementary Fig. S4A (19)), indicating vascular function is a primary target o*f* C/EBPα/β throughout the preovulatory stage between ovulation induction and follicle rupture.

We further categorized all DEGs from *Cebp-C* and *Cebp-P* mutants at 8 hours post hCG into K-means clusters based on their alterations relative to the WT, and performed Gene Ontology (GO) analyses on genes within each cluster (see Fig. 3C). In clusters at 8 hours, 1 and 2, the highest transcript levels of genes were either in WT or in *Cebp-C* mutants, and intermediate levels in *Cebp-P* mutants. Notably, this pattern of transcript levels negatively or positively correlated with the protein levels of C/EBPβ at the same time point (see Fig. 1F), suggesting a dose-dependent regulatory mechanism of C/EBPβ. Genes in these clusters were predominantly associated with BPs related to vascular remodeling. Clusters 8 hours, 3 and 4, consisted of genes that exhibited similar transcript levels in WT and *Cebp-P* mutants but were different in *Cebp-C* mutants, and these genes were involved in ECM organization and vascular remodeling. Dysregulation of genes in clusters 8 hours, 3 and 4, was observed only in *Cebp-C* mutants but not in *Cebp-P* mutants, suggesting that the regulation of their expression depends on C/EBPβ at early preovulatory stages, during which only *Cebp-C* mutants displayed an altered level of C/EBPβ protein compared to WT (0-4 hours post hCG) (see Fig. 1F). In clusters 8 hours, 5 and 6, the transcript levels of genes were either upregulated or downregulated to a similar extent in both mutants compared to the WT, with no statistically significant difference observed between the mutants. This suggests that the regulation of these DEGs depends on the presence of C/EBPα/β during the intermediate preovulatory stages (4-8 hours post hCG) when both mutants exhibited significantly reduced expression levels of C/EBPα/β between 4 and 8 hours post hCG. Genes in those clusters were related to ECM organization and vascular remodeling. The findings from clusters 8 hours, 3 to 6, suggest that C/EBPα/β regulate gene transcription and BPs in a preovulatory stage-specific manner. Clusters 8 hours, 7 and 8, consisted of genes that exhibited upregulation or downregulation exclusively in *Cebp-P* mutants. This pattern of differences is intriguing given that C/EBPα/β were depleted throughout the preovulatory stage in *Cebp-C* mutants but their levels started to reduce only between 4 and 8 hours post hCG in *Cebp-P* mutants compared to WT. It is possible that while transcript levels of these genes changed during 0 to 4 hours post hCG but had returned to the basal level by 8 hours post hCG in WT, they were not changed in response to hCG in the absence of C/EBPα/β as in C*ebp-C* mutants, and they failed to be induced or suppressed between 4 and 8 hours post hCG with reduced C/EBPα/β as in *Cebp-P* mutants. Genes in these clusters were related to oocyte and ovary development and vascular remodeling. Clusters 8 hours, 9 and 10, consisted of genes that exhibited dysregulation in the opposite directions in *Cebp-C* vs *Cebp-P* mutants compared to WT. One plausible explanation for this difference could be that in normal conditions, these genes are suppressed between 0 and 4 hours and then induced between 4 and 8 hours post hCG (or in the reversed order) by C/EBPα/β, and the latter regulation depends on the earlier effect. Genes in these clusters were related to cell-cell adhesion and inflammation. In summary, these findings highlight the stage-specific and dose-dependent roles of C/EBPα/β in regulating gene expression during the preovulatory period, with distinct regulatory mechanisms at different stages and in different mutant contexts. The distinct gene clusters provide insight into how C/EBPα/β orchestrate key BPs such as vascular remodeling, ECM organization, and inflammation during follicular maturation and ovulation.

Next, we applied the same analysis pipeline to the bulk RNA-seq data at 12 hours post hCG. As this is the average time when ovulation occurs in mice, we ensured at the time of sample collection that all samples used were from mice that had not ovulated. At this time point, *Cebp-C* mutants exhibited 341 upregulated and 425 downregulated genes compared to WT, while *Cebp-P* mutants displayed 459 upregulated and 411 downregulated genes (Fig. 3D; Supplementary Table S4 (22)). We identified 186 DEGs overlapping between *Cebp-C* and *Cebp-P* mutants, with 580 DEGs specific to *Cebp-C* mutants and 684 DEGs specific to *Cebp-P* mutants (Fig. 3E). Similar to 8 hours, vascular remodeling–related BPs were enriched in all 3 groups (shared DEG, *Cebp-C*–specific DEG, and *Cebp-P*–specific DEG) (Supplementary Fig. S4B (19)), indicating that vascular remodeling remains a critical process influenced by C/EBPα/β at the time of ovulation. Through K-means clustering followed by GO analyses, we found that at 12 hours post hCG, C/EBPα/β regulate genes in adipogenesis and vascular remodeling in a dose-dependent manner (Fig. 3F, clusters 12 hours, 1 and 2). The trend of difference of genes in these 2 clusters correlated with the rate of ovulation, suggesting that proper regulation of these processes by C/EBPα/β is crucial for successful ovulation. Additionally, vascular remodeling, particularly blood vessel morphogenesis and endothelial cell differentiation, and inflammation-related BPs were specifically affected in *Cebp-C* mutants (see Fig. 3F, clusters 12 hours, 3 and 4). This pattern suggests that C/EBPα/β regulation of these processes occurs mainly before 4 hours post hCG, and the delayed depletion of *Cebpa/b* in *Cebp-P* mutants did not affect these processes at the time close to ovulation, highlighting the timing-dependent regulatory manner of C/EBPα/β in vascular and inflammatory regulation. BPs related to bone development and metabolism were affected in both mutants (see Fig. 3F, cluster 12 hours, 5 and 6), indicating that these processes are broadly regulated by C/EBPα/β throughout the preovulatory stage, regardless of the timing of their depletion. Furthermore, BPs associated with oocyte development and steroidogenesis were affected only in *Cebp-P* mutants (see Fig. 3F, clusters 12 hours, 7 and 8). This suggests that the partial and delayed reduction of C/EBPα/β in *Cebp-P* mutants may be sufficient to disturb these crucial processes, underscoring the sensitivity of oocyte development and steroidogenesis to precise levels of C/EBPα/β.

We further analyzed the DEGs relative to WT that overlap between 8 hours and 12 hours post hCG in *Cebp-C* (Supplementary Fig. S5A (19)) and *Cebp-P* mutant (Supplementary Fig. S5B (19)) to assess the temporal effects of *Cebpa/b* depletion. In *Cebp-C* mutants, 382 DEGs were unique to 8 hours post hCG, 524 DEGs were unique to 12 hours post hCG, and 242 DEGs were consistently present at both time points (see Supplementary Fig. S5A (19)). Blood vessel development–related BPs were enriched across all time points among *Cebp-C*–specific DEGs (at 8 hours, shared DEGs between 8 hours and 12 hours, and at 12 hours post hCG), indicating that vascular function is a primary target of C/EBPα/β throughout the preovulatory stages. These findings suggest that C/EBPα/β regulate vascular remodeling not only with sustained influence, but also through distinct mechanisms at different stages. Similarly, in *Cebp-P* mutants, 1214 DEGs were unique to 8 hours post hCG, 475 DEGs were unique to 12 hours post hCG, and 395 DEGs were consistently present at both time points (see Supplementary Fig. S5B (19)). Blood vessel development–related BPs were also enriched in all groups (at 8 hours, shared DEGs between 8 hours and 12 hours, and at 12 hours post hCG), further supporting the notion that C/EBPα/β directly regulate vascular function from intermediate to late preovulatory stages. These findings reinforce that vascular function is a key target of C/EBPα/β throughout the preovulatory stages, with dynamic transcriptional regulation occurring across different preovulatory stages.

Taken together, these findings suggest that C/EBPα/β regulate gene expression and BPs critical for ovulation through both dose- and preovulatory stage–dependent mechanisms. Moreover, C/EBPα/β regulate the same biological process through different mechanisms at different preovulatory stages. For example, endothelial cell differentiation was enriched in cluster 1 at 8 hours (suggesting dose-dependent regulation) and in cluster 3 in 12 hours (suggesting timing-dependent regulation). In some cases, such as vascular remodeling, multiple regulatory mechanisms may operate simultaneously at the same time point, as indicated by its enrichment across several clusters at 8 hours. These findings reveal the complexity and specificity of the regulatory mechanisms of C/EBPα/β on their downstream targets.

### C/EBPα/β Regulate Ovarian Gene Expression by Dose- and Preovulatory Stage–dependent Mechanisms

**Figure 4.**
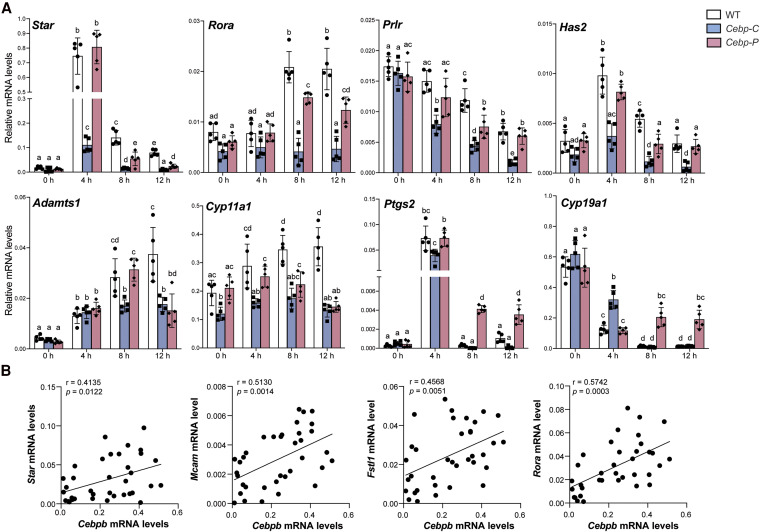
C/EBPα/β regulate ovulation gene expression by dose- and preovulatory stage–dependent mechanisms. A, RT-qPCR shows the mRNA expression levels of selected ovulation-related genes in granulosa cells collected from WT, *Cebp-C*, and *Cebp-P* mutants before and after hCG treatment (n = 5). B, RT-qPCR shows the mRNA levels of selected genes in individual follicles collected from WT and *Cebp-P* mutants at 8 to 9 hours post hCG (n = 3). Quantitative data in this figure are presented as mean ± SD. Two-way ANOVA with Tukey multiple comparisons test was used for statistical analysis in A. Bars without common superscripts are statistically different (*P* < .05). Pearson correlation coefficient was used for correlation analysis in B. *r* and *P* values are indicated in the figure.

Bulk RNA-seq data demonstrate that the downstream targets and mechanism of action (eg, dose-dependency) of C/EBPα/β are highly sensitive and specific to the time window relative to the LH surge and ovulation. To test this idea, we performed RT-qPCR across multiple time points between hCG and ovulation on selected genes identified in various clusters from Fig. 3C and 3F (Fig. 4A). Indeed, several patterns of regulation were observed during the preovulatory stages. First, the mRNA levels of *Star* (regulates cholesterol transport into mitochondria for steroidogenesis (40)) and *Rora* (which plays a role in GC function (42)) increased post hCG in WT but were consistently lower in *Cebp-C* mutants at all time points; at late preovulatory stage (8-12 hours post hCG), their mRNA levels correlated closely with C/EBPβ protein levels across WT, *Cebp-C* mutants, and *Cebp-P* mutants, indicating dose-dependent regulation by C/EBPβ, and that normal levels of C/EBPα/β are necessary for normal mRNA levels of these genes after the early preovulatory stage (0-4 hours post hCG). Second, the mRNA levels of *Prlr* (mediates signaling downstream of prolactin, critical for luteinization (43)) and *Has2* (essential or the production of hyaluronan during cumulus expansion (44)) were lower in *Cebp-C* mutants comparing to WT at all time points post hCG, indicating the importance of C/EBPα/β in the normal expression of these genes. In *Cebp-P* mutants, mRNA levels of these genes were reduced only at 8 hours post hCG, correlating with the protein levels of C/EBPβ, indicating a dose-dependent regulation starting from the intermediate preovulatory stage (∼4-8 hours post hCG). Third, in WT mice, the mRNA levels of *Adamts1* (involved in tissue remodeling during follicle growth (45) and rupture (46)) and *Cyp11a1* (key regulator of steroid hormone production (47)) increased after hCG treatment, and this increase was reduced for *Adamts1* and absent for *Cyp11a1* in *Cebp-C* mutants. Different from *Star*, *Rora*, *Prlr*, and *Has2*, the mRNA levels of *Adamts1* and *Cyp11a1* in *Cebp-P* mutants were at a low level comparable to that of *Cebp-C* mutants by 12 hours post hCG when C/EBPβ protein levels were similarly diminished in both mutants. These observations suggest the induction of *Adamts1* and *Cyp11a1* by hCG is dependent on the dosage of C/EBPα/β in the late preovulatory stage (between 8-12 hours post hCG). Lastly and interestingly, the mRNA levels of *Ptgs2* (a key enzyme for follicle rupture and ovulation (48)) and *Cyp19a1* (which encodes aromatase, responsible for estrogen synthesis (49)) shared strikingly and abnormally high levels in *Cebp-P* mutants compared both to WT and *Cebp-C* mutants at 8 hours post hCG, and the higher levels were sustained until at least 12 hours post hCG. It is intriguing that in the absence of C/EBPα/β protein as in *Cebp-C* mutants, the mRNA levels of *Cyp19a1* and *Ptgs2* dropped to the same low levels as in WT by 8 hours post hCG; the difference between *Cebp-C* and *Cebp-P* mutants therefore may be explained by additional/compensating transcription factors or mechanisms in the absence of C/EBPα/β, or alternatively, suggest that the full dosage or the appropriate LAP/LIP ratio of C/EBPβ during the late preovulatory stage (8-12 hours post hCG) is required for the suppression of these genes. Notably, the mRNA level of *Cyp19a1* was abnormally high in *Cebp-C* mutants compared to WT at 4 hours but not later time points post hCG, suggesting a switch in the mechanism of its transcription suppression between early and late preovulatory stages. Importantly, for all genes assessed in this figure, their mRNA levels were normal in *Cebp-C* mutants at 0 hours post hCG, despite the fact that Cre-mediated recombination had occurred earlier, suggesting that their regulation by C/EBPα/β is primarily after ovulation induction.

We further assessed the correlation between the mRNA levels of DEGs from the aforementioned bulk RNA-seq that are associated with ovulation and mRNA levels of *Cebpb* in isolated individual preovulatory follicles collected from WT and *Cebp-P* mutants at 8 to 9 hours post hCG, which exhibited varied levels of mRNA (see Fig. 2C). We found that the mRNA levels of *Star*, *Mcam* (contributes to ovarian tissue regeneration (50)), *Fstl1* (modulates inflammatory response and matrix remodeling (51)), *Rora*, and *Apln* were significantly correlated with the mRNA levels of *Cebpb* (Fig. 4B, Supplementary Fig. S6 (19)), supporting dose-dependent regulation of these downstream genes by C/EBPβ.

Taken together, these findings demonstrate that C/EBPα/β play a pivotal role in coordinating key ovarian processes, including steroid hormone production, tissue remodeling, follicle rupture, and cumulus expansion, in an expression dosage- and preovulatory stage–dependent manner. The observed varied sensitivity of gene regulation to C/EBPα/β dosage at distinct preovulatory stages highlights the intricacy in gene regulation required for ovulation.

### C/EBPα/β Regulate Vascular Remodeling During the Preovulatory Period

Data from bulk RNA-seq indicate that BPs (eg, vascular development, endothelial cell differentiation, blood vessel morphogenesis) involved in vascular remodeling are among the most affected by the genetic depletion of C/EBPα/β in GCs (see Fig. 3C and 3F and Supplementary Figs. S4 and S5 (19)). As a functional validation of these gene expression data, we examined the structure of the ovarian vascular network in *Cebp-P* mutants at 12 hours post hCG. We reasoned that as the expression of vascular genes in these mutants was dysregulated at least by 8 hours post hCG, subsequent phenotypic abnormalities in ovarian vascular function may be present before ovulation. To test this possibility, we employed a 3D whole-mount imaging method to evaluate ovarian vascular structure immediately before ovulation.

**Figure 5.**
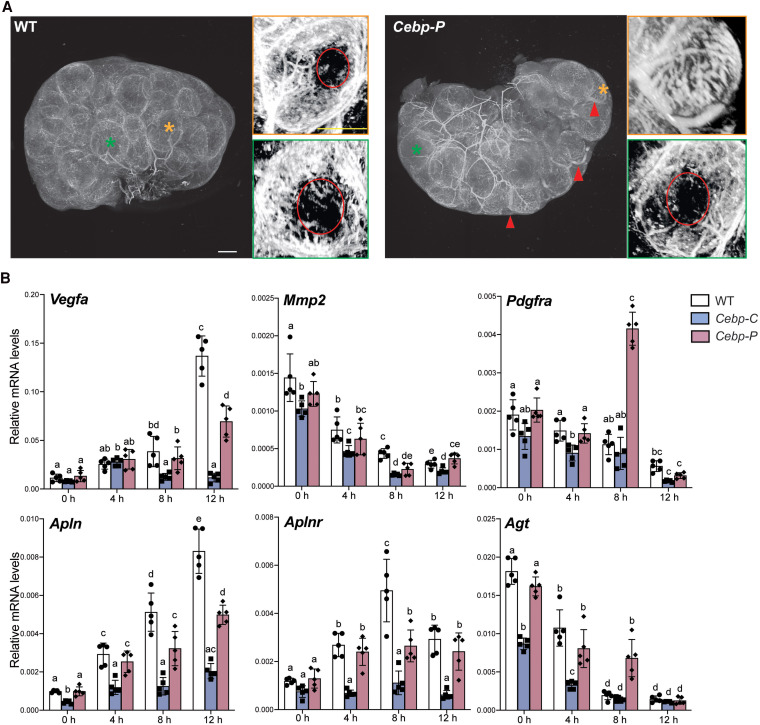
C/EBPα/β regulate ovarian vascular remodeling during the preovulatory period. A, Representative whole-mount 3-D projection images of cleared ovaries with blood vessels labeled by lectin conjugated with Alexa 649 (white) from WT and *Cebp-P* mutants at 12 hours post hCG (n = 3). Colored asterisk: representative preovulatory follicles outlined by the same color. Red arrowhead: abnormal large blood vessels in ovaries of *Cebp-P* mutants that are not observed in WT. Capillary clearing is labeled by a red circle. Scale bar = 250 μm. B, RT-qPCR shows the mRNA expression levels of selected vascular remodeling-related genes in granulosa cells collected from WT, *Cebp-C*, and *Cebp-P* mutants before and after hCG treatment (n = 5). Quantitative data in this figure are presented as mean ± SD. Two-way ANOVA with Tukey multiple comparisons test was used for statistical analysis in B. Bars without common superscripts are statistically different (*P* < .05).

Ovaries from WT and *Cebp-P* mutants were collected after blood vessels were labeled in vivo by Alexa 649–conjugated lectin at 12 hours post hCG (periovulatory). Whole-mount imaging by confocal microscopy was performed after confirming that ovulation had not occurred (Fig. 5A). Ovaries from WT mice displayed homogeneous organization of vascular structure around periovulatory follicles across the ovary (see Fig. 5A, left), with larger blood vessels in the medullary region and in stromal tissues between follicles, and capillary bed surrounding individual follicles. Notably, clearing of the capillary bed at the apex of the periovulatory follicles, which is a vascular remodeling process essential to successful follicle rupture (52), occurred in almost all periovulatory follicles of the WT mice. In contrast, vascular structure varied around individual periovulatory follicles and in different regions across ovaries from the *Cebp-P* mutants (see Fig. 5A, right). Specifically, while in some regions of the ovary, larger blood vessels in the stroma and the clearing of the capillary bed appeared similar to those in the WT mice, in other regions of the ovary there were blood vessels in ovarian stromal tissues with an abnormally large diameter, and the clearing of the capillary bed was absent in some of the periovulatory follicles. Interestingly, periovulatory follicles without capillary clearing were located adjacent to stromal blood vessels with an abnormally larger diameter, suggesting a correlation between the remodeling of stromal blood vessels and the capillary bed. Notably, nearly half of the periovulatory follicles in *Cebp-P* mutants lacked capillary clearing at the apex (Supplementary Fig. S7 (19)), similar to their rate of reduction in ovulation (see Fig. 1C), suggesting the contribution of defective vascular remodeling to impaired ovulation in these mice. Altogether, these observations support data from bulk RNA-seq and indicate C/EBPα/β play a crucial role in regulating the remodeling of both the ovarian stromal blood vessels and apex capillaries in preovulatory follicles.

To understand how C/EBPα/β regulate vascular remodeling during the preovulatory period, we analyzed the expression of vascular remodeling–related genes using RT-qPCR over a series of time points between hCG and ovulation (see Fig. 5B). Similar to ovulation-related genes analyzed in Fig. 4A, temporal patterns of gene expression and disruption were observed. Specifically, the mRNA levels of *Vegfa* increased most robustly between 8 and 12 hours post hCG in WT, and the induction was absent in *Cebp-C* mutants and reduced in *Cebp-P* mutants; in comparison, mRNA levels of *Vegfa* were normal both in *Cebp-C* and *Cebp-P* mutants up to at least 4 hours post hCG. These observations suggest that the basal level of *Vegfa* mRNA is independent of *Cebpa*/*b* in the early preovulatory stages (0-4 hours post hCG), and that the induction of *Vegfa* by hCG is dependent on the dosage of *Cebpa*/*b* in the late preovulatory stage (8-12 hours post hCG). For *Apln*, *Mmp2*, and *Aplnr*, a shared dose-dependent pattern was observed where compared to WT, reduced mRNA levels were detected in *Cebp-C* mutants at all time points, while *Cebp-P* mutants showed reduced mRNA levels primarily at 8 hours post hCG (also at 12 hours post hCG for *Apln*). In contrast, the mRNA levels of *Pdgfra* and *Agt* exhibited abnormally elevated mRNA levels in *Cebp-P* mutants compared both to WT and *Cebp-C* mutants at 8 hours post hCG, and the mRNA levels of these 2 genes were lower in *Cebp-C* mutants compared to WT at 4 hours post hCG (also at 0 hours for *Agt*). This pattern suggests that C/EBPα/β are indispensable for the initial induction and further inhibition of transcription of these genes. Taken together, these findings highlight the gene-specific effects of C/EBPα/β in regulating vascular remodeling at distinct preovulatory stages.

### C/EBPα/β Mediate Gene Regulation in Granulosa Cells via Diverse Mechanisms During the Preovulatory Period

**Figure 6.**
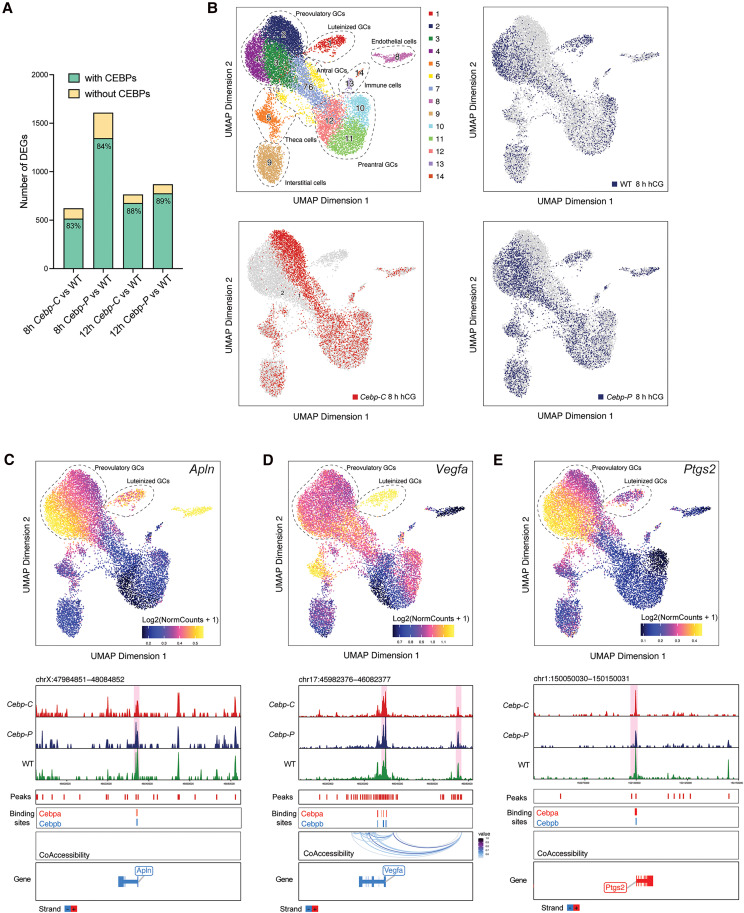
C/EBPα/β mediate gene regulation in granulosa cells via diverse mechanism during the preovulatory period. A, Pan-cistromics analysis shows the proportion of differentially expressed genes in Fig. 3A that contain binding sites for C/EBPα or C/EBPβ within the EPR (TSS-7.5 kb, TSS + 2.5 kb) based on ChIP-seq data from various tissue types. B, UMAPs show the clustering and identification of 14 different cell clusters based on chromatin accessibility from snATAC-seq of ovaries from WT, *Cebp-C*, and *Cebp-P* mutants at 8 hours post hCG. Upper left: combined clustering of all genotypes; upper right: WT; lower left: *Cebp-C*; lower right: *Cebp-P* (WT: n = 2; *Cebp-C*: n = 3; *Cebp-P*: n = 3). C to E, UMAP visualization colored by log2-normalized gene scores, demonstrating the cell cluster-specific chromatin accessibility (top panels) and genome tracks (lower panels) for C, *Apln*; D, *Vegfa*; and E, *Ptgs2* in cell clusters 1 to 4 in as B. Gene scores are calculated as log2(norm counts + 1).

To assess whether transcription regulation of the DEGs identified in *Cebp-C* and *Cebp-P* mutants compared to WT during the preovulatory stage is mediated by C/EBPα/β or indirectly via other mechanisms, we first performed a pan-cistromics analysis. Specifically, we examined in publicly available and ReMap 2022 (35)-validated chromatin immunoprecipitation sequencing (ChIP-seq) data sets from various tissue types whether C/EBPα and C/EBPβ binding sites were present within the EPR (TSS-7.5 kb, TSS + 2.5 kb) of these DEGs. Our analysis revealed that 83% to 89% of DEGs contained at least one binding site for C/EBPα or C/EBPβ (Fig. 6A). Notably, genes such as *Rora*, *Apln*, *Vegfa*, *Adamts1*, and *Ptgs2*, which exhibit dose- and/or preovulatory stage–dependent regulation by C/EBPα/β (see Figs. 4A and 5B), all possess C/EBPα/β binding sites within their EPR (Supplementary Table S6 (36)). These findings suggest that C/EBPα and C/EBPβ have the capacity to bind and directly regulate the expression of the majority of DEGs between mutants and WT, including key genes regulating ovulation.

To further probe how C/EBPα/β regulate gene expression in the ovary, we applied snATAC-seq to whole ovaries obtained from WT, *Cebp-C*, and *Cebp-P* mutants at 8 hours post hCG. Unsupervised clustering revealed 14 major cell clusters that were visualized using UMAP (Fig. 6B, upper left). We annotated the identify of cell clusters based on differentially accessible genes (Supplementary Table S5 (33)) and cell type-specific marker genes for various ovarian cell types (9, 34). The pattern of chromatin accessibility across cell clusters for each genotype were further visualized using UMAP (Fig. 6B, upper right: WT; lower left: *Cebp-C*; lower right: *Cebp-P*). UMAP analysis revealed that the overall pattern of chromatin accessibility was similar between WT and *Cebp-P* mutants but notably different in *Cebp-C* mutants. Specifically, cluster 1 (luteinized GCs) was present in WT and *Cebp-P* but absent in *Cebp-C* mutants. Clusters 3 and 4 (preovulatory GCs) were predominantly found in WT and *Cebp-P* mutants, whereas cluster 2 (preovulatory GCs) was enriched in *Cebp-C* mutants. Given that the depletion of *Cebpa/b* in GCs of *Cebp-C* mutants starts during follicle development before the preovulatory stage (16), whereas *Cebp-P* mutants retain normal C/EBPα/β expression until at least 4 hours after hCG administration (see Fig. 1E and 1F), the aberrant distribution of chromatin accessibility across GC clusters in *Cebp-C* mutants indicates the importance of C/EBPα/β during the early preovulatory stage or possibly earlier in guiding the proper differentiation trajectory of GCs. Together, these findings support a model in which C/EBPα/β regulate chromatin accessibility in a stage-dependent manner, playing a particular critical role during the early preovulatory stage or earlier to establish the chromatin landscape necessary for transcriptional regulation during ovulation.

We next examined chromatin accessibility of selected genes whose transcript levels were previously characterized as C/EBPα/β dose- or preovulatory stage–dependent. Browser plots were generated to visualize chromatin accessibility at gene annotation regions within clusters 1 to 4, which represent luteinized and preovulatory GC populations most relevant to ovulatory function, across WT, *Cebp-C*, and *Cebp-P* mutants (see Fig. 6C-6E, Supplementary Fig. S8A and S8B (19)). At *Apln*, whose mRNA levels are regulated by C/EBPα/β in a dose-dependent manner at 8 hours post hCG, there was moderate chromatin accessibility in luteinized GCs (cluster 1) that was absent in *Cebp-C* mutants, but high chromatin accessibility in preovulatory GCs (clusters 2-4) that were predominant in WT and *Cebp-P* mutants. In contrast, chromatin accessibility decreased in regions shifting toward the cluster predominant in *Cebp-C* mutants (see Fig. 6C, upper panel). Browser plot of chromatin accessibility at gene annotation regions within ovulation-related GC population (clusters 1-4) further showed that chromatin accessibility at the *Apln* promoter region was highest in WT, intermediate in *Cebp-P*, and lowest in *Cebp-C* (see Fig. 6C, lower panel). This pattern closely paralleled mRNA expression levels of *Apln* across genotypes at 8 hours post hCG (see Fig. 5B). We then overlapped the differentially accessible regions (DARs) identified at the *Apln* locus with remapped C/EBPα and C/EBPβ binding sites from ReMAP2022 within the EPR of *Apln* (see Fig. 6C, lower panel, Supplementary Table S7 (37)). This analysis revealed that the published C/EBPα and C/EBPβ binding sites overlapped with the DARs at the *Apln* promoter, suggesting that C/EBPα/β may directly bind to the promoter of *Apln* and modulate its chromatin accessibility and transcriptional regulation. These findings suggest that C/EBPα/β regulate *Apln* expression by modulating its chromatin accessibility and transcriptional regulation in a dose-dependent manner. *Rora*, whose mRNA levels are also regulated by C/EBPα/β in a dose-dependent manner, exhibited a similar overall pattern of cell cluster–specific chromatin accessibility as *Apln* (Supplementary Fig. S8A, top panel (19)). However, chromatin accessibility at the *Rora* promoter region was highest in WT, while *Cebp-C* and *Cebp-P* mutants displayed similar levels of accessibility (see Supplementary Fig. S8A, lower panel (19)). Notably, there were no overlapping DARs and C/EBPα and C/EBPβ binding sites at the *Rora* promoter based on currently available ChIP-seq data sets in the pan-cistromics analysis. This suggests that the transcriptional regulation of *Rora* at 8 hours post hCG may involve additional or indirect mechanisms. However, with the limitation that ovary-specific ChIP-seq data are unavailable and our snATAC seq (8 hours post hCG) data have limited temporal resolution, we cannot exclude the possibility of direct C/EBPα/β binding occurring at this or other stages of the preovulatory period.

*Vegfa* exhibited high chromatin accessibility in luteinized GCs (cluster 1), while preovulatory GCs (clusters 2-4) showed moderate chromatin accessibility with no notable differences among clusters (see Fig. 6D, upper panel). Browser plot showed that chromatin accessibility at the *Vegfa* promoter region and putative enhancer regions was similar between WT and *Cebp-P*, but reduced in *Cebp-C* mutants (see Fig. 6D, lower panel). This pattern is consistent with its mRNA expression levels, which were comparable between WT and *Cebp-P*, but significantly lower in *Cebp-C* mutants at 8 hours post hCG (see Fig. 5B). In contrast, *Adamts1* showed low chromatin accessibility in luteinized GCs (cluster 1) but high chromatin accessibility in preovulatory GCs (clusters 2-4), particularly in clusters predominant in WT and *Cebp-P* mutants, whereas cluster predominate in *Cebp-C* mutants exhibited moderate chromatin accessibility (see Supplementary Fig. S8B, top panel (19)). Browser plot showed that promoter accessibility of *Adamts1* was similar between WT and *Cebp-P*, but again lowest in *Cebp-C* mutants (see Supplementary Fig. S8B, lower panel (19)). This pattern also mirrors the transcript levels of *Adamts1*, which were comparable in WT and *Cebp-P* but significantly reduced in *Cebp-C* mutants at 8 hours post hCG (see Fig. 4A). Importantly, DARs overlapped with C/EBPα and C/EBPβ binding sites within the EPR both of *Vegfa* and *Admats1* (see Fig. 6D and Supplementary Fig. S8B (19), lower panel, and Supplementary Table S7 (37)). Given that both promoter chromatin accessibility and transcript levels of *Vegfa* and *Adamts1* were comparable between WT and *Cebp-P* mutants at 8 hours post hCG, but reduced in *Cebp-C* mutant, these findings suggest that C/EBPα/β likely serve as direct transcriptional regulators of these genes during the early preovulatory stage (0-4 hours post hCG), when *Cebp-P* mutants still maintain *Cebpa/b* levels similar to WT. Their binding may help establish or maintain an open chromatin state at key loci, thereby facilitating transcriptional activation during the preovulatory period.

*Ptgs2*, which is regulated by C/EBPα/β in a manner that does not appear dose- or preovulatory stage–dependent, exhibited moderate chromatin accessibility in luteinized GCs (cluster 1) and high chromatin accessibility in preovulatory GCs (clusters 2-4), particularly in clusters 3 and 4, which are predominant in WT and *Cebp-P* mutants. In contrast, cluster 2, which predominates in *Cebp-C* mutants, displayed moderate chromatin accessibility (see Fig. 6E, top panel). Browser plot showed that chromatin accessibility at the *Ptgs2* promoter region was highest in WT, intermediate in *Cebp-C*, and lowest in *Cebp-P* mutants, with DARs that overlapped with C/EBPα and C/EBPβ binding sites within the EPR (see Fig. 6E, lower panel, Supplementary Table S7 (37)). This pattern appears contradictory to its mRNA expression levels, which were significantly increased in *Cebp-P* mutants at 8 hours post hCG (see Fig. 4A). These findings suggest an alternative regulatory mechanism for *Ptgs2* in *Cebp-P* mutants, for which C/EBPα/β may be required to maintain promoter accessibility for effective recruitment of repressive transcriptional regulators. In the context of reduced C/EBPα/β levels during the intermedia preovulatory stage (4-8 hours post hCG), as seen in *Cebp-P* mutants, reduced chromatin accessibility at the *Ptgs2* promoter may paradoxically reflect a failure to recruit transcriptional repressors, resulting in aberrant gene activation. This is consistent with findings that chromatin accessibility does not always correlate with active transcription (53, 54) and that effective gene repression may also rely on an open chromatin state to permit binding of inhibitory factors (55).

Taken together, these observations indicate that C/EBPα/β modulate global chromatin accessibility in GCs up to the early preovulatory stage. For downstream target genes, C/EBPα/β may directly regulate their transcription by promoter binding, while for others, C/EBPα/β may maintain an open chromatin state that facilitates interactions with additional transcription factors, including repressors. These mechanisms are likely gene specific and reflect the context-dependent roles of C/EBPα/β in preovulatory gene regulation.

## Discussion

This study supports the crucial role of preovulatory C/EBPα/β in directly regulating ovulation after the LH surge/hCG. We find that sustained expression of C/EBPβ throughout the preovulatory stage is necessary for normal ovulation, and the dose of *Cebpa/b* positively correlates with the outcome of ovulation. We identified BPs involved in vascular remodeling as a primary functional target of C/EBPα/β downstream of the LH surge. Furthermore, our data revealed that the mechanism by which C/EBPα/β regulate ovulation is sensitive and specific to the level of their expression, the time windows/stages between hCG/LH and ovulation, and the downstream genes/BPs. Specifically, our data suggest that C/EBPα/β regulate chromatin accessibility in GCs during the early preovulatory stage. At later preovulatory stages, C/EBPα/β may regulate gene transcription by direct binding to the regulatory regions of target genes or by maintaining an open chromatin state that facilitates access for other transcription factors and coregulators.

We demonstrate that C/EBPα/β directly regulate ovulation during the preovulatory stage, and their effect on ovulation depends on the level of their expression (dose-dependent effect), in particular that of C/EBPβ. Previous research showed that both global and GC-specific depletion of *Cebpb* lead to compromised ovulation (15, 56). However, the precise mechanisms underlying ovulatory failure in both cases were not clearly defined. In *Cebp-C* mutants, GC-specific depletion of *Cebpa*/*b* occurs through Cre recombinase driven by the promoter of *Cyp19a1* (14), which becomes active during the antral follicle stage (16). This raises the question of whether ovulation failure in *Cebp-C* mutants results primarily from *Cebpa/b* depletion before the LH surge or from their direct regulatory roles during the preovulatory stage. The impaired ovulation observed in the *Cebp-P* mutants, in which the depletion of *Cebpa/b* occurs after hCG/LH stimulation, indicates that C/EBPα/β directly regulate ovulation. In particular, our data suggest that sustained expression of *Cebpb* throughout the preovulatory stages is essential for successful ovulation. At the whole ovary level, the extent of *Cebpa/b* depletion negatively correlated with the rate of ovulation, as seen when comparing *Cebp-C* and *Cebp-P* mutants. At the individual follicle level, ovulation outcome was closely associated with the expression levels of *Cebpb* (see Fig. 2A and Supplementary Fig. S3 (19)). These in vivo observations underscore a dose-dependent regulatory effect of *Cebpb* on ovulatory efficiency, which aligns with findings that employed an ex vivo ovary perfusion system demonstrating a correlation between *Cebpb* level and ovulation rate (57).

Our data reveal intricate mechanisms by which C/EBPα/β regulate gene expression in preovulatory follicles, including dose- and preovulatory stage–dependent mechanisms (see Fig. 3). In clusters 1 and 2 in Fig. 3C and 3F, the levels of gene expression are correlated with the levels of C/EBPβ protein. A similar dose-dependent mechanism of regulation by C/EBPβ has been documented in the liver (13). C/EBPβ has also been shown to regulate gene expression in a stage-dependent manner in other tissues, such as during liver regeneration (58), where it regulates genes related to acute phase response in early stages and genes involved in metabolic and DNA synthesis during the later stages of regeneration. In clusters 3 and 4 in Fig. 3C and 3F, genes unaffected in *Cebp-P* mutants indicate that their regulation by C/EBPα/β occurs during the early preovulatory stages. Conversely, genes affected both in *Cebp-C* and *Cebp-P* mutants suggest a need for sustained levels of C/EBPβ for their regulation. Interestingly, by 8 hours post hCG, *Cebpa* levels do not differ between WT and *Cebp-P* mutants, suggesting that the genes unaffected in *Cebp-P* mutants at this time point might be regulated by C/EBPα rather than by C/EBPβ (clusters 3 and 4 in Fig. 3C). Genes in these clusters are involved in vascular remodeling and ECM organization, processes known to be critical for ovulation (3, 45). However, considering that *Cebpa^gc−/−^* females exhibit no significant reduction in ovulation rate, whereas *Cebpb^gc−/−^* females show a significant reduction in ovulation (14), it is more likely that these genes are regulated by *Cebpb*, contrasted with the subtler effects of *Cebpa*. The lower rate of ovulation seen in the *Cebpa/b^gc−/−^* double-knockout models suggests a potential hierarchical or compensatory relationship between the 2 transcription factors, where C/EBPβ serves as the primary regulator, but C/EBPα may fine-tune gene expression during the preovulatory stage. Future studies are needed to clarify the direct downstream effects of *Cebpa* and *Cebpb*, and the interaction between them in coordinating gene regulation during the preovulatory period.

Some genes exhibit patterns of regulation by C/EBPα/β that cannot be explained solely by dose- or preovulatory stage–dependent mechanisms. These genes show exclusive upregulation or downregulation in *Cebp-P* mutants without the same trend of change in *Cebp-C* mutants. In addition to differences in dose and depletion timing of *Cebpa/b* among WT, *Cebp-C*, and *Cebp-P* mutants, we also observed alterations in the ratio of LAP to LIP isoforms of C/EBPβ in *Cebp-P* mutants (see Supplementary Fig. S2C (19)). C/EBPβ has been shown to have both activating and inhibitory effects through different isoforms (13), and the regulatory mechanism of C/EBPβ is influenced by the ratio of its LAP and LIP isoforms (59). We found that in addition to decreased C/EBPβ protein levels, the LAP/LIP ratio was increased in *Cebp-P* mutants at 8 hours post hCG compared to WT (see Supplementary Fig. S2C (19)). While the exact effect of this shift in LAP/LIP ratio remains to be determined, it raises the possibility that the genes dysregulated exclusively in *Cebp-P* mutants may be influenced by this shift or by potential interaction between C/EBPα with LAP/LIP complexes, which could form heterodimers favoring transcriptional activation (11, 60). Further investigation is needed to test these mechanisms.

*Cebp-P* mutants exhibit abnormal ovarian vascular remodeling in areas of stromal tissue surrounding the periovulatory follicles that lack proper capillary clearing (see Fig. 5A). This phenotype of vascular abnormality aligns with our findings that C/EBPα/β regulate vascular-related genes in a dose-dependent manner (see Figs. 3C and 3F and 4B). The consistent association between *Cebpa/b* expression levels and the regulation of vascular remodeling processes underscores the importance of C/EBPα/β in maintaining proper ovarian vascular function. Furthermore, the varied levels of *Cebpa/b* depletion in individual preovulatory follicles in *Cebp-P* mutants at the late preovulatory stages may contribute to impaired ovulation by affecting vascular remodeling processes crucial for ovulation.

C/EBPα/β are crucial both for the induction and repression of key genes such as *Ptgs2*, *Agt*, and *Pdgfra* in response to the LH surge, exhibiting a biphasic regulatory pattern (see Figs. 4A and 5B). Specifically, between 0 and 4 hours post hCG, C/EBPα/β are necessary for the induction of these genes, while between 8 and 12 hours post hCG, C/EBPα/β appear to actively suppress their transcription (see Fig. 4A and 5B). This biphasic mode of gene regulation—characterized by an initial induction phase followed by a suppression phase—highlights the complex temporal dynamics that govern ovulation. Despite the recognition of this complexity, little is known about the mechanisms that turn off gene expressions during the late preovulatory stage. For example, genes such as *Ptgs2* and *Pdgfra* exhibit biphasic regulation, where their transcription is initially induced but later suppressed by full dosage of C/EBPα/β (see Figs. 4A and 5B). Our work addresses this critical knowledge gap and sheds light on how C/EBPα/β orchestrate the precise timing modulation of gene expression necessary for successful ovulation. Insights from this work underscore the importance of understanding these gene regulatory mechanisms in greater detail.

Our study highlights the critical role of C/EBPα/β in regulating chromatin accessibility during the preovulatory period. C/EBPα/β are known to play a role in chromatin remodeling to facilitate or restrict access to promoters of target genes (61). For example, C/EBPβ has been recognized as a pioneering transcription factor in adipose tissue, where it initiates dynamic changes in the chromatin landscape and recruits other transcription factors within the first hour of adipocyte differentiation (62). Our findings suggest a similar role in the ovary, where C/EBPα/β (predominantly C/EBPβ) establish chromatin landscape during early preovulatory stage that is necessary for the proper differentiation of GCs (see Fig. 6B). Our data from snATAC-seq analyses demonstrate that C/EBPα/β influence chromatin accessibility in a dose- and preovulatory stage–dependent manner in GC clusters undergoing luteinization and preovulatory differentiation. The differential chromatin accessibility patterns observed between *Cebp-C* and *Cebp-P* mutants further highlight the stage-specific functions of C/EBPα/β in orchestrating the transcriptional dynamics of ovulation. Notably, genes involved in regulating chromatin remodeling are actively regulated as early as 0 to 1 hour post hCG (7), supporting the idea that chromatin landscapes are established during the early preovulatory stage and C/EBPα/β are required at this early stage to initiate chromatin remodeling necessary for proper transcriptional regulation. Furthermore, genes essential for angiogenesis (*Vegfa*, *Apln*), follicle rupture (*Adamts1*), and GC function (*Rora*) exhibit distinct chromatin accessibility patterns in WT, *Cebp-C*, and *Cebp-P* mutants, and these patterns are correlated with the level of transcripts for these genes, indicating that C/EBPα/β regulate their transcriptional activity via modulating chromatin accessibility. Interestingly, our findings suggest that C/EBPα/β-mediated chromatin accessibility does not always directly correlate with levels of transcripts for specific genes, such as *Ptgs2*, which showed an inverse relationship between mRNA expression and chromatin accessibility across 3 genotypes (see Figs. 4A and 6E). This phenomenon has been observed previously, where chromatin accessibility does not always equate to active transcription (53, 54). In some cases, open chromatin may instead facilitate the binding of transcriptional repressors (55). These observations suggest that in addition to direct DNA binding and transcriptional activation, C/EBPα/β may also contribute to maintaining promoter accessibility and enable the recruitment of repressive transcription factors, thereby modulating both activation and inhibition of gene transcription during the preovulatory stage.

The capacity of C/EBPα/β to exert opposing effects during different preovulatory stages may stem from their interactions with distinct transcription factors and coactivators. In other tissues, C/EBPα/β have been documented to interact with RUNX1 and RUNX2 to regulate gene expression (63, 64). In the ovary, RUNX1 binds to the promoter region of *Ptgs2* during the early preovulatory stage, facilitating *Ptgs2* induction in response to the LH surge (63). Conversely, RUNX2—rather than RUNX1—binds to the same promoter to inhibit *Ptgs2* expression at later preovulatory stages (65). Thus, C/EBPα/β may collaborate with RUNX1/2 at different stages to finely tune the dynamics in gene expression throughout the preovulatory period, influencing both the activation and repression of critical genes like *Ptgs2*. The differential roles of C/EBPα/β in regulating *Ptgs2* transcription at various ovulatory stages may involve a complex relationship between chromatin accessibility and gene transcription. Indeed, during the intermediate preovulatory stage (4-8 hours post hCG), *Ptgs2* expression is actively suppressed in WT, yet its promoter region exhibits the highest chromatin accessibility in WT. In contrast, *Cebp-P* mutants display the lowest chromatin accessibility at *Ptgs2* promoter but highest mRNA expression levels at 8 hours post hCG. These findings imply that C/EBPα/β may be required to maintain high chromatin accessibility, allowing repressive transcription factors, such as RUNX2 (65), to control the timing dynamics of *Ptgs2* expression. Taken together, our data suggest that the effects of C/EBPα/β on chromatin accessibility during early preovulatory stages may predetermine the timing of gene regulation during later preovulatory stages. Further investigation into this potential mechanism is warranted to fully understand how C/EBPα/β orchestrate complex gene regulation during ovulation.

In conclusion, our data demonstrate that C/EBPα/β regulate ovulation and preovulatory vascular remodeling in a dose-dependent manner. In line with this function, C/EBPα/β regulate the expression of genes involved in key BPs essential for ovulation and luteinization, such as GC luteinization, angiogenesis, ECM remodeling, and inflammation. The mechanism by which C/EBPα/β regulate these processes depends on the dose of *Cebpa/b* and/or the stages during the preovulatory period, highlighting the complexity in gene regulation during ovulation. This work also sheds light on the mechanism regulating the temporal dynamics of gene expression during the preovulatory period, exemplified by the finely tuned activation and suppression of *Ptgs2*. Given these intricate effects of C/EBPα/β in the regulation of ovulation, disruption in the function of C/EBPα/β may contribute to impaired ovulation, and manipulating the activity of C/EBPα/β could offer new venues for contraception design as well as therapeutic interventions to improve ovulation.

## Data Availability

The authors declare that the bulk RNA sequencing data supporting the findings of this study have been deposited in NCBI's Gene Expression Omnibus (GEO) with GEO series accession number GSE279171. The snATAC-sequencing data that support the finding of this study are available from the corresponding author on reasonable request. Supplemental figures and supplemental tables can be found at the following site and accessed via the following links (19, 21, 22, 33, 36, 37): https://doi.org/10.6084/m9.figshare.28726718 https://doi.org/10.6084/m9.figshare.28726733 https://doi.org/10.6084/m9.figshare.28726724 https://doi.org/10.6084/m9.figshare.28726721 https://doi.org/10.6084/m9.figshare.28726727 https://doi.org/10.6084/m9.figshare.28726730.

## Abbreviations

3D: 3-dimensional
BP: biological process
BSA: bovine serum albumin
C/EBPα/β: CCAAT/enhancer-binding proteins α and β
ChIP-seq: chromatin immunoprecipitation sequencing
CL: corpus luteum
CRM: cis regulatory module
DAR: differentially accessible region
DEG: differentially expressed gene
ECM: extracellular matrix
EPR: extended promoter region
GC: granulosa cell
GO: Gene Ontology
hCG: human chorionic gonadotropin
LAP: liver-enriched activator protein
LH: luteinizing hormone
LIP: liver-enriched inhibitory protein
mRNA: messenger RNA
PBS: phosphate-buffered saline
PTGS2: prostaglandin-endoperoxide synthase 2
RNA-seq: RNA sequencing
RT-qPCR: real-time quantitative polymerase chain reaction
snATAC-seq: single-nucleus assay for transposase-accessible chromatin sequencing
TPM: transcripts per million
TR: transcriptional regulator
TSS: transcription start site
UMAP: uniform manifold approximation and projection
VEGFA: vascular endothelial growth factor A
